# Are there systematic differences in Teacher Assessment and Test Scores by Exclusion Status?

**DOI:** 10.23889/ijpds.v9i5.2686

**Published:** 2024-09-18

**Authors:** Neil Rowland, Duncan McVicar, Babak Jahanshahi, Corina Miller

**Affiliations:** 1 Queen's University Belfast

## Objective and Approach

The educational performance of school pupils in England is assessed in many ways, including through formal testing and teacher assessment. It is important to understand whether certain types of pupils perform better in certain forms of assessment, and although this has been studied within the context of ethnicity, amidst a recent increase in the number of school exclusions in England there is a growing need to understand whether excluded pupils perform differently than never-excluded pupils in different forms of assessment, especially given that excluded pupils are characterized by multiple forms of disadvantage. This study investigates this question using the Longitudinal Educational Outcomes (LEO) dataset, focusing on a cohort of pupils who undertook both forms of assessment at the end of Key Stage 3 (age 14).

## Results

We first explore whether, compared with never-excluded pupils, excluded pupils receive a teacher assessment level below their achieved test level, conditional on prior attainment. These estimates reveal no differences by exclusion record, and this result holds for all assessed subjects (English, Maths and Science). We also explore whether, conditional on the Key Stage 3 test score, excluded pupils have a higher chance of being assessed below the expected level by their teacher. These estimates indicate that, compared with measurably similar pupils who are never excluded, some excluded pupils underachieve in teacher assessment.

## Conclusions and Implications

Overall, the results show that what can be learned about systematic discrepancies between teacher assessment and test measures depends on how such discrepancies are estimated.

